# Rv1717 Is a Cell Wall - Associated β-Galactosidase of *Mycobacterium tuberculosis* That Is Involved in Biofilm Dispersion

**DOI:** 10.3389/fmicb.2020.611122

**Published:** 2021-01-15

**Authors:** Suman Bharti, Rahul Kumar Maurya, Umamageswaran Venugopal, Radhika Singh, Md. Sohail Akhtar, Manju Yasoda Krishnan

**Affiliations:** ^1^Microbiology Division, CSIR-Central Drug Research Institute, Lucknow, India; ^2^Toxicology and Health Risk Assessment Division, CSIR-Indian Institute of Toxicology Research, Lucknow, India; ^3^Molecular and Structural Biology Division, CSIR-Central Drug Research Institute, Lucknow, India

**Keywords:** galactosidase, mycobacteria, biofilm, biofilm dispersion, extracellular polymeric substance

## Abstract

Understanding the function of conserved hypothetical protein (CHP)s expressed by a pathogen in the infected host can lead to better understanding of its pathogenesis. The present work describes the functional characterization of a CHP, Rv1717 of *Mycobacterium tuberculosis* (Mtb). Rv1717 has been previously reported to be upregulated in TB patient lungs. Rv1717 belongs to the cupin superfamily of functionally diverse proteins, several of them being carbohydrate handling proteins. Bioinformatic analysis of the amino acid sequence revealed similarity to glycosyl hydrolases. Enzymatic studies with recombinant Rv1717 purified from *Escherichia coli* showed that the protein is a β-D-galactosidase specific for pyranose form rather than the furanose form. We expressed the protein in *Mycobacterium smegmatis* (Msm), which lacks its ortholog. In Msm*^Rv1717^*, the protein was found to localize to the cell wall (CW) with a preference to the poles. Msm*^Rv1717^* showed significant changes in colony morphology and cell surface properties. Most striking observation was its unusual Congo red colony morphotype, reduced ability to form biofilms, pellicles and autoagglutinate. Exogenous Rv1717 not only prevented biofilm formation in Msm, but also degraded preformed biofilms, suggesting that its substrate likely exists in the exopolysaccharides of the biofilm matrix. Presence of galactose in the extracellular polymeric substance (EPS) has not been reported before and hence we used the galactose-specific *Wisteria floribunda* lectin (WFL) to test the same. The lectin extensively bound to Msm and Mtb EPS, but not the bacterium *per se*. Purified Rv1717 also hydrolyzed exopolysaccharides extracted from Msm biofilm. Eventually, to decipher its role in Mtb, we downregulated its expression and demonstrate that the strain is unable to disperse from *in vitro* biofilms, unlike the wild type. Biofilms exposed to carbon starvation showed a sudden upregulation of *Rv1717* transcripts supporting the potential role of Rv1717 in Mtb dispersing from a deteriorating biofilm.

## Introduction

*Mycobacterium tuberculosis* (Mtb) caused nearly 10.0 million new tuberculosis (TB) cases and 1.5 million TB deaths in the year 2018 (WHO, 2019). Mtb is a highly successful pathogen because of its ability to persist in the host despite a functional immune system. The persister population though is a small percentage, can survive the antibiotic exposure and necessitate the lengthy treatment of tuberculosis and pose a significant challenge to TB control (Zhang et al., 2012). Development of Mtb persisters, despite extensive research, is still inadequately understood. Mtb H37Rv genome contains approximately 4,000 genes (Cole et al., 1998), of which approximately 25% of protein-coding genes is annotated as hypothetical proteins (HPs), which are proteins that have no assigned function (Doerks et al., 2012; Mazandu and Mulder, 2012; Yang et al., 2019). HPs can be unique for a pathogen and potentially important for its pathogenicity. Therefore, characterizing the HPs of Mtb and discovering their role in its virulence or persistence will guide the development of novel strategies to counter this deadly pathogen. Identification and characterization of the proteins involved in persistence will aid developing novel drugs for shorter anti-tubercular regimens. The transcriptional response of sputum Mtb that survive the early bactericidal phase of anti-TB therapy for drug susceptible TB suggested that drug-tolerant bacilli in sputum are slow growing and metabolically downregulated (Walter et al., 2015). Analysis of Mtb transcription pattern in serial sputa helps to shortlist those proteins that are upregulated specifically after 14 days of treatment. These proteins could be important for the bacilli to persist amidst therapy.

Rv1717 is a small (116 amino acids) conserved hypothetical protein (CHP) that was highly upregulated in 90% of the patients after 14 days of anti-tubercular treatment (Walter et al., 2015). Rv1717 is thought to be expressed by the non-replicating bacilli in TB sputa (Garton et al., 2008). The gene has 100% identical homologues in all sequenced clinical isolates of Mtb, *Mycobacterium bovis*, and *M. bovis* BCG. Orthologues are also present in many non-tubercular mycobacteria, other actinobacteria, and proteobacteria. However, it is notably absent in some other pathogenic mycobacteria for instance, the *Mycobacterium avium* complex, *Mycobacterium marinum*, and *Mycobacterium leprae*. The protein has a cupin domain, a conserved domain characteristic of the cupin super family of proteins, the members of which are spread across Archaea, Eubacteria, and Eukaryota (Dunwell, 1998). This extremely diverse superfamily includes catalytically inactive seed storage and sugar-binding metal-independent proteins as well as metal-dependent enzymes like dioxygenases, decarboxylases, and others (Uberto and Moomaw, 2013). The 3D structures of the enzymatic and non-enzymatic members gave them the collective name cupins on the basis of their β-barrel shape (Latin term “cupa,” small barrel). The characteristic β-barrel is made up of two conserved motifs, each consisting of two β-strands, separated by a less conserved region composed of another two β-strands with an intervening variable loop (Dunwell et al., 2004).

The present work aims to understand the molecular and cellular function of Rv1717 in Mtb. First, we over expressed the protein in the *Escherichia coli* host and used the purified protein to determine its molecular function. For deciphering its cellular function, we expressed it in *Mycobacterium smegmatis* that lacks an ortholog. We show that Rv1717 is a β-D-galactosidase localized on the cell wall (CW). It is involved in cleaving galactose or N-Acetylgalactosamine containing exopolysaccharide in the extracellular polymeric substance (EPS). Further, we demonstrate that a Mtb strain with downregulated expression of Rv1717 shows impaired dispersion from *in vitro* biofilms.

## Materials and Methods

### Bacterial Strains, Growth Conditions, Primers, and Plasmids

*Escherichia coli* DH5α was used as a host strain for cloning and plasmid propagation, while the C41 (DE3) strain was used for recombinant protein expression and purification. *Escherichia coli* was propagated in Luria-Bertani (LB) broth (HiMedia Laboratories, Mumbai), at 37°C with continuous shaking. *M. smegmatis* mc^2^155 and *M. tuberculosis* (H37Rv and H37Ra strains) were cultured in Middlebrook (MB) 7H9 broth medium supplemented with 10% oleic acid-albumin-dextrose-catalase (OADC; Becton Dickinson, Franklin Lakes, NJ), 0.05% tween 80 and 0.2% glycerol. MB 7H10 agar medium (BD) was supplemented with 10% OADC and 0.5% glycerol. Kanamycin (kan) or Ampicillin (amp) was added to all media at a final concentration of 30 μg/ml and 100 μg/ml respectively whenever required. *E. coli* expression vector pET21d was used from lab stock, while *E.coli*-mycobacteria shuttle vector pMV261 was a kind gift from William R. Jacobs, Jr. (Department of Microbiology and Immunology, Albert Einstein College of Medicine, New York, NY). All primers used in the study are listed in Supplementary Table S1.

### Overexpression and Purification of Recombinant Rv1717

The *Rv1717* coding sequence 351 bp was amplified from Mtb H37Rv genomic DNA by PCR using Taq DNA polymerase (NEB, MA, United States) and cloned into expression vector pET21d (+; Novagen) between the NheI and XhoI restriction sites. The overexpression strain *E. coli* C41 (DE3) was cultured in 5 ml of LB broth up to OD_600_ ∼ 0.4–0.6. Expression was induced with 0.2 mM isopropyl β-D-1-thiogalactopyranoside (IPTG) and the culture was further incubated for overnight at 30°C under 140 rpm shaking condition. Overexpression of the protein in the lysate was analyzed by 15% SDS-PAGE and confirmed by western blot using HRP-conjugated anti-His antibody (Santa Cruz Biotechnology, TX, United States). Optimal solubilization was achieved by the addition of urea (final conc. of 4 M) to the lysis buffer.

For purification, 2 L of culture were induced with 0.2 mM IPTG for overnight at 30°C. Cells were harvested, resuspended in lysis buffer containing 4 M urea and sonicated at 20 s on/off cycle for 30 min. The supernatant was loaded into the column containing pre-equilibrated Ni-nitrilotriacetic acid (Ni-NTA) agarose beads (Qiagen, Hilden, Germany) and incubated for 1–2 h at 4°C. After incubation it was washed serially with 100 ml of wash buffer [50 mM Tris-HCl (pH 8.0), 300 mM NaCl, and 10% glycerol]. Each 100 ml of wash buffer contained decreasing concentration of urea (3–0 M) and increasing concentration (20–100 mM) of imidazole. The protein was eluted with elution buffer [50 mM Tris-HCl (pH 8.0), 300 mM NaCl, 10% glycerol and 1,000 mM imidazole]. The fractions containing Rv1717 protein were pooled and protein purity was checked by SDS-PAGE. The protein was dialyzed in three MWCO membrane (Thermo Fisher Scientific, MA) against 500 ml dialysis buffer [50 mM Tris-HCl (pH 8.0), 300 mM NaCl, and 10% glycerol] containing decreasing concentration of imidazole (800–200 mM) at 4°C. The concentration of purified Rv1717 protein was determined by Bradford colorimetric method by using Bradford reagent (HiMedia Laboratories, Mumbai, India).

### Circular Dichroism Spectroscopy

Far-UV circular dichroism (CD) spectra were used to quantify native state and secondary structure content of refolded purified Rv1717 protein. The spectra were acquired on a Jasco J810 polarimeter. The spectra were measured in the range of 5–20 μM protein dialyzed against phosphate buffer pH 8.0 containing 50 mM NaCl. Far-UV CD spectra measurements were recorded from 190 to 240 nm at a scan speed of 10 nm/min at 25°C. The obtained values were normalized by subtracting the baseline recorded for the buffer. The CD result was expressed as mean residue ellipticity (MRE) in degree cm^2^dmol^−1^.

### Enzyme Assay

Enzyme activity was determined as described elsewhere (Michikawa et al., 2012) with some modifications, using the various p-nitrophenol (pNP)-glycosides as substrates. pNP-β-D-glucopyranoside (pNP-β-D-Glu), pNP-β-D-galactopyranoside (pNP-β-D-Gal), pNP-β-D-mannopyranoside (pNP-β-D-Man), pNP-α-L-arabinopyranoside (pNP-α-L-Ara), pNP-α-L-rhamnopyranoside (pNP-α-L-Rha), pNP-β-D-glucopyranosiduronic acid (pNP-β-D-GlcA), and pNP-α-D-galactopyranoside (pNP-α-D-Gal) were purchased from Sigma-Aldrich (St. Louis, MO, United States). pNP-β-D-galactofuranoside (pNP-β-D-Gal*f*) and pNP-2-acetamido-2-deoxy-β-D-galactopyranoside (pNP-β-D-GalNAc) were purchased from Santa Cruz Biotechnology (Dallas, TX, United States) and GLR innovations (New Delhi, India) respectively. Enzymatic reaction was performed in a 200 μl mixture containing 1 mM pNP-glycoside and 1.8 μM of Rv1717 protein in 100 mM Na_2_HPO_4_ (pH 7.0) buffer. The reactions were carried out at 37°C for 1 h and terminated by the addition of 50 μl of 0.2 M Na_2_CO_3_. Enzyme activity was determined by monitoring the amount of the pNP released from pNP-glycosides at 405 nm. One unit (U) of enzyme activity was defined as the amount of enzyme that released 1 μmol of pNP from pNP-glycosides per minute under the standard assay condition. The enzyme activity values were presented as the mean values of triplicate assays.

### Enzyme Assay at Different Temperature, pH, or Metal Ions

To determine the optimum temperature, standard enzyme reactions containing 0.36 μM purified Rv1717 protein and 1 mM of pNP-β-D-Gal were performed in sodium phosphate buffer pH 8.0 at different incubation temperatures such as 10, 20, 30, 37, 40, 50, 60, 70, and 80°C. For the determination of the optimum pH, standard enzyme reaction containing 0.36 μM purified Rv1717 protein were performed using sodium phosphate buffer of different pH from 3 to 10, at 37°C. For each temperature and pH, a blank sample (enzyme reaction without substrate) was used as control. A plot of relative activity against different temperature and pH values was created. All enzyme assays were performed in triplicates and reported as mean values ± SD.

The effect of metal ions on the activity of Rv1717 protein was performed in the presence of various metal ions of BaCl_2_ (Ba^2+^), CdSO_4_ (Cd^2+^), MgCl_2_ (Mg^2+^), MnCl_2_ (Mn^2+^), FeCl_2_ (Fe^2+^), CaCl_2_ (Ca^2+^), CoCl_2_ (Co^2+^), NiSO_4_ (Ni^2+^), CuSO_4_ (Cu^2+^), ZnCl_2_ (Zn^2+^), or chelating agent ethylenediaminetetraacetic acid (EDTA) at a final concentration of 0.5 mM. After pre-incubation of various metal ion or EDTA with 0.28 μM purified Rv1717, the reaction mixtures were further incubated with pNP-β-D-Gal for 1 h at 37°C. The enzyme activity without any additional reagents was considered to be 100% and relative activity of the enzyme in presence of the metal ion was calculated.

### Determination of Enzyme Kinetic Parameters

Enzyme assay was performed with recombinant Rv1717 using different concentrations of pNP-β-D-Gal (0–10 mM) in sodium phosphate buffer (pH 8.0) at 37°C for 1 h. The values of the kinetic constants were calculated using the Michaelis-Menten method. The initial data were plotted as initial velocities V° (micromolar of pNP released) vs. [S] (millimolar of substrate used). Kinetic parameters were calculated by fitting the values calculated from secondary plots of inverse of reaction velocity (1/V°) vs. inverse of substrate concentration (1/[s]) to the Lineweaver-Burk equation 1/V = (Km/Vmax×[S]) + 1/Vmax using GraphPad Prism software.

### Construction of Recombinant Mycobacterial Strains

Recombinant *M. smegmatis* mc^2^155 strains, Msm*^Rv1717^* (expressing Rv1717 with C-terminal 6 × His-tag) and Msm^pMV261^ (empty vector): The *Rv1717* ORF (351 bp) was amplified by PCR from Mtb H37Rv genomic DNA using forward and reverse primers containing EcoRI and HindIII restriction enzyme sites respectively (Supplementary Table S1). The amplicon containing six consecutive His codons at the 3′-end of the ORF sequence was cloned into pMV261 vector (episomal) harboring the *hsp65* constitutive promoter. Empty vector (pMV261) and recombinant plasmid (pMV261-*Rv1717*) were electroporated into *M. smegmatis* mc^2^155. Recombinant strain Msm*^Rv1717^* and Msm^pMV261^ were selected on supplemented MB7H10 agar containing 30 μg/ml kan.

Msm*^rfp^* and Msm*^Rv1717-rfp^*: The coding sequence for FusionRed (Evrogen, Moscow, Russia), a monomeric red fluorescent protein, was synthesized after codon optimization for mycobacteria (Integrated DNA Technologies, Coralville, IA, United States). This sequence was cloned either alone or fused to the 3′ end of Rv1717 coding sequence through a 36 bp sequence (encoding a 12 residue flexible linker) in to pMV261 vector. The DNA fragments for fusion were generated by PCR and cloned into pMV261 vector through fusion cloning using In-Fusion(R) HD cloning kit (TakaraBio, CA, United States). The confirmed constructs (pMV261-*rfp* and pMV261-*Rv1717-rfp*) were extracted from *E. coli* DH5α and electroporated into *M. smegmatis* mc^2^155.

Rv1717 knock-down mutant strain of *M. tuberculosis* H37Rv: The 351 bp *Rv1717* coding sequence was amplified by PCR from Mtb H37Rv genomic DNA using forward and reverse primers containing HindIII and EcoRI restriction enzyme sites respectively (Supplementary Table S1). The PCR product was cloned in the inverse orientation into pMV261 vector. The recombinant construct (pMV261-*KDRv1717*) was confirmed by restriction digestion prior to electroporation in Mtb H37Rv. Recombinant strain Mtb*^KDRv1717^* and Mtb^pMV261^ were selected on supplemented MB7H10 agar containing 30 μg/ml kan.

### Colony Morphology

Cultures of Msm*^Rv1717^* and Msm^pMV261^ of OD_600_ ∼ 0.5 were declumped by syringing with 26G needle to generate single cell suspensions. The cultures were diluted in MB7H9 broth medium and 10 μl was spotted onto supplemented MB7H10 + kan agar plates. Plates were incubated at 37°C for 3–5 days or till colonies matured. Colonies were visualized using 4 × objective under a Nikon eclipse E100 microscope and photographed.

### Congo Red Dye Assay

The assay was performed according to reported methodology (Sonden et al., 2005). Five milliliters cultures of both Msm*^Rv1717^* and Msm^pMV261^ strains were grown in MB7H9 broth to OD_600_ ∼ 0.6–0.8. Cultures were sonicated to break the clumps and increase the homogeneity. Cultures were centrifuged at 4,700 × g for 15 min, the pellets were washed with phosphate buffered saline-tween 80 (0.05% v/v; PBST) and resuspended in the same to an OD_600_ ∼ 0.6–0.8. Two microlitres of cell suspensions were spotted on MB7H9 medium supplemented with 1.5% agar, 100 μg/ml Congo red, 0.02% glucose, and 30 μg/ml kan and incubated for 3–5 days at 37°C. Colony morphology and staining were examined under the Nikon eclipse E100 microscope and photographed.

### Growth Curve

Msm*^Rv1717^* and Msm^pMV261^ were grown in 5 ml MB7H9 broth complete medium containing 0.05% tween 80 and kan upto mid-log phase under shaking condition at 37°C. These cultures were used to inoculate fresh medium at an OD_600_ of 0.01. The optical density was measured at regular intervals up to 60 h of incubation at 37°C. Growth curve was plotted with OD_600_ values (in triplicates) against time.

### Ethidium Bromide Accumulation and Nile Red Uptake Assays

Msm*^Rv1717^* and Msm^pMV261^ were grown in MB7H9 broth medium containing kan to mid-log phase culture, washed twice with PBST and resuspended in 2 ml of PBST. The bacterial suspension was adjusted to OD_600_ ∼ 0.5 and 200 μl was added in triplicates to 96-well black clear bottom plates. Ethidium bromide (EtBr) accumulation and Nile red uptake were quantified as described elsewhere (Chuang et al., 2015), with minor modifications. EtBr or Nile red was added to the cultures at a final concentration of 1 μg/ml or 20 μM, respectively. The uptake/accumulation of dye was measured as relative fluorescence intensity at every 5 min for 1 h duration using multimode microplate reader (SpectraMax M2, Molecular Devices, CA, United States) at excitation 530 nm and emission 590 nm.

### Sodium Dodecyl Sulfate Sensitivity Assay

Sodium dodecyl sulfate (SDS) sensitivity assay was carried out according to a previously described method (Chen et al., 2013). Cultures were grown in MB7H9 + 0.05% tween 80 + kan broth to an OD600 ∼ 0.2. Cultures were pelleted and washed with PBST and resuspended at a density of ~1.5 × 10^8^ cells/ml in MB7H9 broth containing no SDS or SDS (0.05 or 0.1% w/v). The cultures were incubated for 3 and 6 h at 37°C in shaking incubator. After incubation cells were harvested, washed twice with PBST and resuspended in original volume of PBST. Cultures were serially diluted in PBST and CFU counts were estimated on MB7H10 + kan agar plates.

### Subcellular Fractionation of *Mycobacterium smegmatis* and Immunoblotting

The cultures of Msm^pMV261^ and Msm*^Rv1717^* strains were grown up to mid-log phase (OD_600_ ∼ 0.4–0.6). Cells were harvested by centrifugation at 8000 × *g* for 30 min. Cell pellets were resuspended in ice cold PBS containing 1 mM PMSF, 1× protease inhibitor cocktail, DNase, and RNase. Cells were lysed in a high pressure cell disruptor (Constant Systems Ltd., Northants, United Kingdom) two times at 20000 lb. in^−2^. The whole cell lysate (WCL) was centrifuged at 3,000 × *g* to remove the unbroken cells. Crude CW, cell membrane (CM), and cytosolic fractions (CY) were obtained by differential ultracentrifugation as described elsewhere (Mawuenyega et al., 2005). The protein concentration in each fraction was estimated by Bradford protein assay. Equal amount (30 μg) of each fraction was separated by 15% SDS-PAGE and transferred onto PVDF membrane (Merck, NJ, United States). The membrane was blocked with 5% skimmed milk in 1 × PBS at room temperature for 1 h. The membrane was further incubated with primary antibodies [anti-6 × His-tag mouse monoclonal antibody (1: 5000) (Santa Cruz Biotechnology, Dallas, TX, United States; anti-mycobacterial Hsp65 mouse monoclonal antibody (Santa Cruz Biotechnology, Dallas, TX, United States; 1: 1000), and anti-*M. smegmatis* LAM monoclonal antibody NR-13798 (BEI Resources, Manassas, VA; 1: 200)], washed with PBS containing tween 20 (0.05%) and incubated with anti-mouse secondary antibody-HRP (1:5000; Santa Cruz Biotechnology, Dallas, TX, United States) in 5% BSA in PBST for 1–2 h. Membrane was washed with PBST before incubating with the Luminol Reagent (Santa Cruz Biotechnology, Dallas, TX). Imaging of the blot was done using a ChemiDoc system (Bio-Rad, CA, United States).

### Biofilm Biomass Quantification

Biofilm formation assay was carried out as described elsewhere (Ojha et al., 2008) with slight modifications. Briefly, the *M. smegmatis* strains (wild type, Msm*^Rv1717^* or Msm^pMV261^) strains were grown in MB7H9 + OADC + glycerol + tween 80 ± kan broth to mid-log phase. This culture was used to inoculate complete Sauton’s medium (Ojha et al., 2015) without tween 80 to an OD_600_ ∼ 0.03. This culture was distributed as 2 ml/well into 12-well plates or 0.2 ml/well into 96-well plates in triplicates. In experiments with wild type *M. smegmatis*, Rv1717 purified protein (0.01–5 μM) or sodium phosphate buffer was added to each well to a final volume of 200 μl. The plates were incubated statically at 37°C in CO_2_ incubator for biofilm formation for 3–5 days at 37°C. Biofilms were quantified by crystal violet assay as described elsewhere (Pang et al., 2012). Briefly, the biofilms were dried at 37°C and incubated with 200 μl of 1% (w/v) crystal violet for 10 min. Wells were washed three times with sterile PBS to remove the unbound dye and dried again. Around 200 μl of 95% ethanol was added to each well and incubated for 10 min. Then biofilm biomass was quantified spectrophotometrically at A_595_ after making 2-fold serial dilutions in case of 12 well plates or directly in case of 96-well plates.

### Biofilm Disruption Assay

Five hundred microliters of Rv1717 protein (5 μM) were added to wild type *M. smegmatis* mature biofilms in Sauton’s medium. Sodium phosphate buffer pH 8.0 was used as the negative control. The vials were incubated for 24 h statically at 37°C. After incubation, the vials were washed with sterile PBS. The biofilm was quantified by crystal violet assay.

### Aggregation Assay

This assay was performed as described elsewhere (Yang et al., 2017) with slight modification. Briefly, the cultures of Msm*^Rv1717^* and Msm^pMV261^ were grown until saturation in 10 ml of complete MB7H9 medium without tween 80. Cultures were centrifuged at 6,000 × *g* for 10 min, cell pellets were washed and resuspended in 2 ml of PBS. The cellular aggregates in the suspension were broken by repeated passage through a 26G needle fitted to a syringe and OD_600_ was adjusted to 1. One milliliter of this suspension was used for OD_600_ measurements at 1 min interval for 10 min in a spectrophotometer (SpectraMax M2, Molecular Devices, CA, United States). Aggregation index scores were calculated using the following equation:

$$\mathrm{Aggregation Index}=\left({\mathrm{OD}}_{T=0}-{\mathrm{OD}}_{T=t}\right)\times 100/{\mathrm{OD}}_{T=0}$$

### EPS Extraction and Purification

Biofilms of wild type *M. smegmatis* were prepared as described above. After development of a thick biofilm, EPS extraction was carried out as previously described (Bales et al., 2013). The crude EPS obtained was filtered through a 0.2 μm filter and dialyzed against distilled water using a 10 kDa MWCO membrane for 24 h at 25°C. Exopolysaccharides were purified from the EPS extract essentially by a procedure described by Bales et al. (2013). The resultant ethanol precipitated exopolysaccharide was resuspended in Milli-Q and dialyzed in PBS using a 10 kDa MWCO membrane for 24 h at 4°C.

### WFL-Exopolysaccharide Binding Assay

Purified exopolysaccharide was coated onto the wells of ELISA plates (50 μl/well) by incubation overnight at 37°C. After incubation, uncoated exopolysaccharides were removed by extensive washing with wash buffer [10 mM Tris-HCl (pH 7.5), 10 mM CaCl_2_, 250 mM NaCl, and 0.05% (w/v) tween 20]. Fifty microliters of purified Rv1717 protein (12 μM) in activity buffer/buffer alone were added to coated wells and incubated for 1 h at 37°C. Plates were washed five times with wash buffer and further incubated with 50 μl *Wisteria floribunda* lectin (WFL)-fluorescein (100 μg/ml; Vector laboratories, CA, United States) for 1 h at 37°C. Plates were washed five times and the fluorescence was recorded in a multimode microplate reader (SpectraMax M2, Molecular Devices, CA, United States) at 495 nm excitation and 515 nm emission.

### Ring Biofilm Dispersion Assay

The ring biofilm dispersion method described elsewhere (Stacy et al., 2016) was used with slight modifications. Briefly, Mtb^pMV261^ and Mtb*^KDRv1717^* strains were grown in complete MB7H9 broth containing kan and tween 80 to mid-log phase. This culture was used to inoculate Sauton’s medium without tween 80 to an OD_600_ ∼ 0.03. Five milliliters were aliquoted into 50 ml conical polypropylene tubes and incubated under shaking condition at 37°C to form ring biofilms. After formation of ring biofilm, medium was removed and tubes were washed 4–5 times with 1× PBS to remove loosely attached biofilm and planktonic cells. Double the original volume of fresh Sauton’s medium with or without glycerol was added to the tubes and further incubated under shaking at 37°C for 36 h. Biofilms were photographed after staining with crystal violet (0.1% w/v).

### Microtiter Plate Biofilm Dispersion Assay

Biofilm dispersion assay was performed in 12-well microtiter plate by a method described elsewhere (Stacy et al., 2014) with slight modifications. Briefly, Mtb^pMV261^ and Mtb*^KDRv1717^* cultures of OD_600_ ∼ 0.03 in Sauton’s medium with kan and without tween 80 were aliquoted into a 12-well microtiter plate containing sterile coverslips. The plates were incubated statically at 37°C. After the formation of biofilm, medium was removed, coverslips were retrieved and washed thrice with 1× PBS to remove loosely attached cells. Coverslips were transferred to a fresh 12-well microtiter plate and incubated in Sauton’s medium with or without glycerol statically at 37°C for 6 h. Biofilm biomass after 6 h was estimated by crystal violet assay as described before.

### Confocal Laser Scanning Microscopy

Single cell suspensions of bacteria were prepared by syringing before applying to coverslips. Planktonic cultures were harvested by centrifugation and washed twice with PBST before applying to coverslips. Biofilms were grown in Sauton’s medium in 12-well polyvinyl chloride (PVC) plates containing sterile coverslips. After the development of biofilms, the coverslips were retrieved and washed with sterile PBS. Depending on the experiment, the coverslips were stained with 100 μM BacLight™ Green bacterial stain (Invitrogen, CA, United States) or 50 μg/ml Fluorescein-tagged WFL (Vector Laboratories, CA, United States) and fixed in 4% formalin. The stained coverslips were examined and imaged in a Leica TCS SP5 confocal laser scanning microscope (Leica, Wetzlar, Germany) or Zeiss LSM 880 (Jena, Germany) confocal laser scanning microscope.

### RNA Extraction and Quantitative Reverse Transcriptase PCR

Total RNA was extracted from planktonic cultures or biofilm of Mtb using RNeasy mini kit (Qiagen, Hilden, Germany) as described previously (Rastogi et al., 2016). RNA was quantified by using Nano Drop Spectrophotometer (Thermo scientific Massachusetts, United States) followed by DNase I (Thermo scientific Massachusetts, United States) treatment for 1 h at 37°C in order to remove DNA contamination prior to cDNA synthesis. Around 1 μg of total RNA sample was reverse transcribed in 20 μl reaction volume using Go Script™ Reverse Transcriptase cDNA Synthesis Kit (Promega, Wisconsin, United States) following manufacturer’s protocol. Control reactions, lacking reverse transcriptase, were performed for every sample. The product of cDNA synthesis was used directly in qRT-PCR. Primers used for qRT-PCR are listed in Supplementary Table S1. qPCR amplification conditions comprised of standard cycle of Go Script™ DNA polymerase activation at 95°C for 10 min, 40 cycles of denaturation at 95°C for 20 s, annealing and extension at 60°C for 1 min. The specificity of the PCR products was verified by agarose gel electrophoresis and melting curve analysis. Results were normalized with a *SigA* gene as endogenous control and calculated by using 2^−*Δ*ΔCT^ method (Livak and Schmittgen, 2001).

### Data Analysis

Data analysis was performed using GraphPad Prism version 5.01 (San Diego, CA, United States). One-way or two-way ANOVA with Bonferroni’s post tests was used to determine the significance of observed differences. Differences were considered statistically significant when *p* was <0.05, <0.01, or <0.001, denoted by ^*^, ^**^, or ^***^, respectively.

## Results

### Purified, Recombinant Rv1717 Protein Shows β-D-Galactosidase Activity

**Figure 1:**
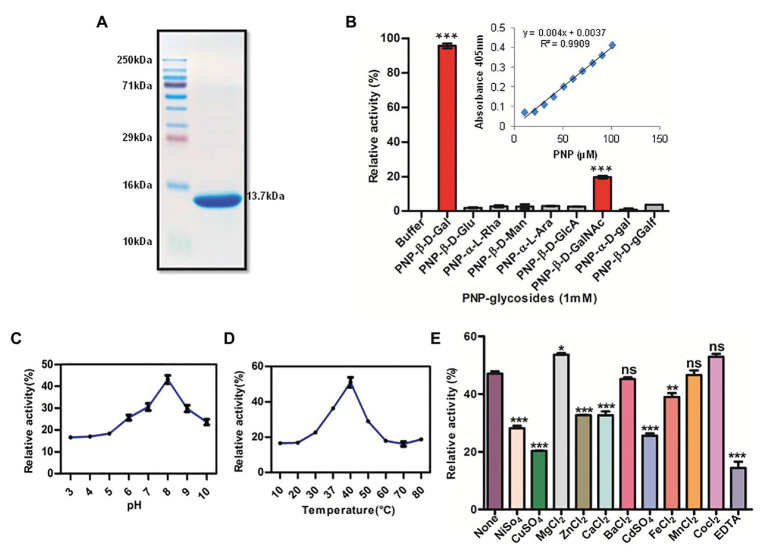
Recombinant Rv1717 purified from *Escherichia coli* shows β-galactosidase activity. **(A)** SDS-PAGE of His-tagged Rv1717 protein (13.7 kDa) purified by Ni-nitrilotriacetic acid (Ni-NTA) affinity chromatography. **(B)** Glycosyl hydrolase activity of Rv1717 was tested using a set of p-nitrophenyl (pNP)-glycosides, pNP-β-D-glucopyranoside (pNP-β-D-Glu), pNP-β-D-galactopyranoside (pNP-β-D-Gal), pNP-β-D-mannopyranoside (pNP-β-D-Man), pNP-α-L-arabinopyranoside (pNP-α-L-Ara), pNP-α-L-rhamnopyranoside (pNP-α-L-Rha), pNP-β-D-glucopyranosiduronic acid (pNP-β-D-GlcA), pNP-α-D-galactopyranoside (pNP-α-D-Gal), pNP-β-D-galactofuranoside (pNP-β-D-Gal*f*), and pNP-2-acetamido-2-deoxy-β-D-galactopyranoside (pNP-β-D-GalNAc) as substrates. One unit (U) of enzyme activity was defined as the amount of enzyme that released 1 μmol of p-nitrophenol (pNP) from pNP-glycosides per minute under the standard assay condition. Graph shows the enzymatic activity of Rv1717 obtained for various substrates relative to the buffer without the protein. Inset shows the standard curve of pNP used to calculate the enzymatic release of pNP. **(C,D)** The effect of pH and temperature respectively, on the enzymatic activity with pNP-β-D-Gal substrate. **(E)** Effect of adding metal ions or EDTA (final concentration of 0.5 mM) to the enzymatic reaction with pNP-β-D-Gal substrate. Control reaction contains none of these. Data plotted are mean ± SD of three independent experiments.

For molecular characterization, Rv1717 was overexpressed with a C-terminal histidine tag in *E. coli* C41 (DE3) strain. Majority of the overexpressed protein (13.7 kDa) was found insoluble, which was solubilized by 4 M urea. The protein was purified by Ni-NTA chromatography, which yielded approximately 0.2–0.3 mg/ml of pure protein (Figure 1A). Refolding of the protein to a stable native structure was confirmed by far-UV circular dichroism (Supplementary Figure S1).

A bioinformatic search of Rv1717 in the Kyoto Encyclopedia of Genes and Genomes (KEGG) database collection revealed that in addition to the cupin domain, Rv1717 contains a glycoside hydrolase (GH) motif. According to the Carbohydrate Active Enzymes database or CAZy (Lombard et al., 2014) definition, glycosyl hydrolases (GHs/glycosidases) are enzymes that catalyze the hydrolysis of the glycosidic linkage of glycosides, leading to the formation of a sugar hemiacetal or hemiketal and the corresponding free aglycon. The GH activity was tested using various p-Nitrophenyl (pNP) glycosides as chromogenic substrates in 100 mM sodium phosphate buffer pH 7.0. The primary screening was done with pNP-β-D-Glu, pNP-β-D-Gal, pNP-β-D-Man, pNP-α-L-Ara, pNP-α-L-Rha, and pNP-β-D-GlcA. GH activity was considered directly proportional to the amount of pNP released. Rv1717 protein showed the highest activity for p-Nitrophenyl-β-D-galactopyranoside (pNP-β-Gal) and only negligible activity for other substrates. The galactosidase activity was specific for the β-glycosidic bond and the pyranoside isomer, since activity was negligible for pNP-α-D-Gal and pNP-β-D-Gal*f*. However, moderate activity was obtained for pNP-2-acetamido-2-deoxy-β-D-galactopyranoside (N-acetylgalactosamine). The results, summarized in Figure 1B, clearly indicate that Rv1717 is a β-D-galactosidase of Mtb that specifically recognizes the β-glycosidic bonds formed with galactose or N-acetylgalactosamine.

The optimum pH and temperature for the enzymatic activity of Rv1717 protein were determined using pNP-β-Gal as the substrate over a range of pH 3–10 and temperature 10–80°C in 100 mM sodium phosphate buffer under the standard assay conditions. The enzymatic activity was maintained over a pH range of 6–10 and temperature range of 30–50°C. Maximum activity was found at pH 8.0 and 40°C (Figures 1C,D). Various divalent metals or EDTA were tested for potential enhancing or inhibitory effect on the enzymatic activity. The metal cations (Ba^2+^, Ca^2+^, Cd^2+^, Fe^2+^, Mg^2+^, Mn^2+^, Ni^2+^, Cu^2+^, Co^2+^, and Zn^2+^) or EDTA were added at final concentrations of 0.5 mM to the standard enzymatic reaction. Only Mg^2+^ was found to enhance the β-galactosidase activity, while Ca^2+^, Cd^2+^, Fe^2+^, Ni^2+^, Cu^2+^, and Zn^2+^ significantly inhibited the enzyme activity. Ba^2+^, Co^2+^, and Mn^2+^ did not affect the activity significantly. However, the addition of EDTA inhibited enzyme activity (Figure 1E).

For determination of kinetic parameters, the reaction mixture was incubated at 37°C for 30 min or up to that time the rate increases linearly. Michaelis-Menten constants for pNP-β-D-Gal were determined by varying its concentration from 0.1 to 10 mM. The initial data were plotted as initial velocities V° (microgram of released pNP per min) vs. [S] (millimoles of substrate). Kinetic parameters were calculated by fitting the values calculated from secondary plots (1/V°) vs. inverse of substrate concentration (1/[S]) to the Lineweaver-Burk equation 1/V° = (K_m_/V_max_ × [S]) + 1/V_max_ using GraphPad Prism software (Supplementary Figure S2). The K_m_ and V_max_ values corresponding to pNP-β-D-Gal for Rv1717 protein was determined to be 0.382 mM and 157.3 μM/min respectively.

At present the CAZy database lists 167 GH families and their subfamilies. Since Rv1717 contains RmlC-like jelly roll/Cupin domain, the amino acid sequence was aligned with multiple sequences from GH families having β-jelly-roll structure like GH7, GH11, GH12, GH16, and GH54. However, no significant alignments with known active site motifs were found. Moreover, Rv1717 is not among the various proteins of *M. tuberculosis* H37Rv classified into various CAZy families. Hence, Rv1717 does not belong to any existing GH family in the CAZy database.

### Overexpression of Rv1717 in *Mycobacterium smegmatis* Changes Its Colony Morphology, Cell Wall Permeability and Sensitivity to Cell Wall Perturbing Agent

**Figure 2:**
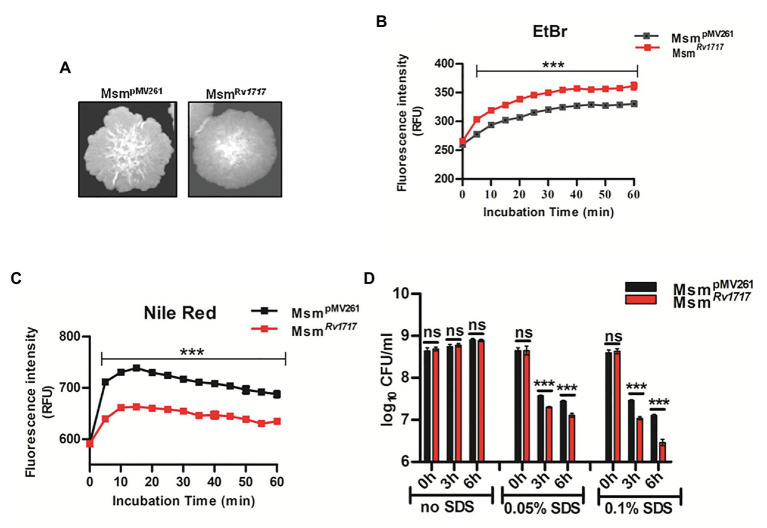
*Mycobacterium smegmatis* expressing Rv1717 shows altered colony morphology and cell wall (CW) associated properties. **(A)** On MB 7H10 agar, *M. smegmatis* harboring empty vector (Msm^pMV261^) formed rough colonies with irregular margins, while *M. smegmatis* expressing Rv1717 (Msm*^Rv1717^*) formed smoother colonies with more regular margins. **(B)** Accumulation of EtBr and **(C)** Nile red by Msm^pMV261^ and Msm*^Rv1717^* **(D)** Msm*^Rv1717^* and Msm^pMV261^ were left untreated or treated with 0.05 or 0.1% (w/v) Sodium dodecyl sulfate (SDS) for 3 and 6 h. Viable cells in all samples were estimated by CFU assay. Statistical significance of data wherever applicable is indicated by ns: *p* > 0.05; ^***^p < 0.001. Data plotted are mean ± SD of three independent experiments.

Comparative genomic analysis showed that Rv1717 does not have any homolog in the non-pathogenic species *M. smegmatis*. Hence to get clues regarding its probable function in Mtb, we overexpressed Rv1717 with a C-terminal 6 × His tag in *M. smegmatis* mc^2^155, which is often used as a surrogate host strain for characterizing Mtb proteins (Snapper et al., 1990). The overexpression of Rv1717 protein was confirmed by detection of the His-tagged protein (12.7 kDa) on western blot using anti-His-tag antibody (Supplementary Figure S3). Cultures of Msm*^Rv1717^* and Msm^pMV261^ strains (empty vector control) were allowed to form single colonies on MB7H10 + kan agar plates. After 5 days of growth, the colonies of Msm*^Rv1717^* were smoother with round margins compared to the colonies of Msm^pMV261^ (Figure 2A). Both strains showed similar growth rates in broth medium (Supplementary Figure S4). The visible difference in colony morphology prompted us to examine the CW associated properties like permeability and surfactant stability.

Uptake of EtBr (hydrophilic) and that of Nile red (lipophilic) have been used to assess cell wall permeability in mycobacteria (Chuang et al., 2015; Yan et al., 2017). EtBr, when free in the medium is less fluorescent, but becomes strongly fluorescent when intercalated to DNA, which also inhibits its extrusion from the cell (Rodrigues et al., 2011). Following preincubation with EtBr, Msm*^Rv1717^* accumulated EtBr in significantly higher amounts compared to Msm^pMV261^ (Figure 2B). Nile red shows weak fluorescence in aqueous solutions but becomes strongly fluorescent in non-polar environments and is widely used for staining intracellular lipids (Greenspan et al., 1985). Unlike in the case of EtBr, Msm*^Rv1717^* strain showed significantly lower uptake of Nile red compared to the Msm^pMV261^ strain (Figure 2C). Overall, the observations suggest altered cell wall permeability following overexpression of Rv1717 in *M. smegmatis*.

Next, the stability to the cell wall perturbing detergent was tested by treating Msm^pMV261^ and Msm*^Rv1717^* strains with different concentrations of SDS (0, 0.05, or 0.1% w/v) for 3 and 6 h. In the absence of SDS, both strains grew at similar rate, as evident from the similar viable counts at 3 and 6 h. SDS at 0.05 and 0.1% reduced the viable counts of either strain by ≥10-fold (1 log_10_). However, Msm*^Rv1717^* was more sensitive to SDS compared to Msm^pMV261^ control strain (*p* < 0.001), suggesting that overexpression of Rv1717 increased the susceptibility toward the surface stress due to SDS (Figure 2D). To summarize, overexpression of Rv1717 in *M. smegmatis* significantly alters its cell wall associated properties, like colony morphology, permeability and surfactant sensitivity.

### Rv1717 Shows Polar Localization in the Cell Wall of *Mycobacterium smegmatis*

**Figure 3:**
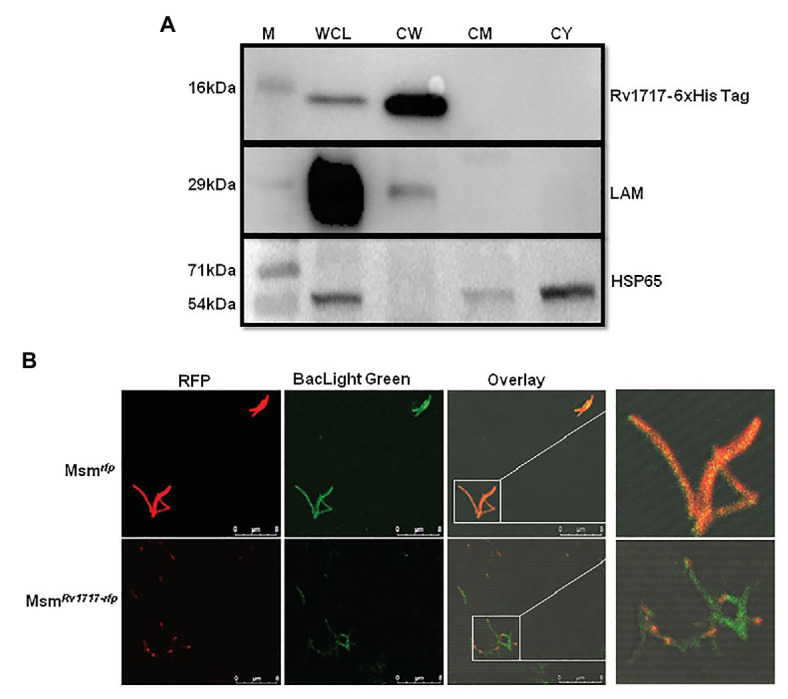
Rv1717 expressed in *M. smegmatis* localizes to the CW and prefers the poles. **(A)** Western blot analysis of equal amounts (30 μg protein) of whole cell lysate (WCL), CW, cell membrane (CM), and cytosolic (CY) fractions of Msm*^Rv1717^*. Rv1717 which was expressed with a C-terminal 6 × His tag was detected by anti-6 × His tag antibody. Lipoarabinomannan (LAM) and Hsp65 were detected using anti-*M. smegmatis* LAM monoclonal antibody NR-13798 (BEI Resources, Manassas, VA) and anti-Hsp65 monoclonal antibody, respectively. LAM was used as marker for the cell wall, while Hsp65 was used as major cytosolic marker. **(B)** A monomeric RFP was expressed in *M. smegmatis* either alone (Msm*^rfp^*) or as a C-terminal fusion to Rv1717 (Msm*^Rv1717-rfp^*). Both the strains were stained with BacLight Green^(^™^)^ fluorescent dye and subjected to confocal microscopy. Individual fluorescence of RFP and BacLight Green^(^™^)^ were recorded and merged. Unfused RFP fluorescence was distributed uniformly across the bacterium, while Rv1717-RFP fluorescence was localized more toward one of the poles.

The amino acid sequence of Rv1717 does not have the classical signal sequences required for secretion through Sec, Tat, lipoprotein, or ESX protein secretion systems, and hence, we performed sub-cellular localization of the His-tagged protein in the cell lysate of Msm*^Rv1717^*. The C-terminal 6 × His-tag in the protein was detected on western blots by anti-His-tag antibody. Rv1717 was detected in the cell wall fraction (Figure 3A). Additionally, we tried to locate the protein by fusing a monomeric red fluorescent protein to the C-terminus of Rv1717 and over expressing the same in *M. smegmatis* (Msm*^Rv1717-rfp^*). Confocal imaging showed that the fusion protein localizes more towards the poles. Control strain carrying the RFP alone showed red fluorescence across the whole cell length (Figure 3B).

### Expression of Rv1717 in *Mycobacterium smegmatis* Significantly Affects Biofilm Formation and Congo Red Staining

**Figure 4:**
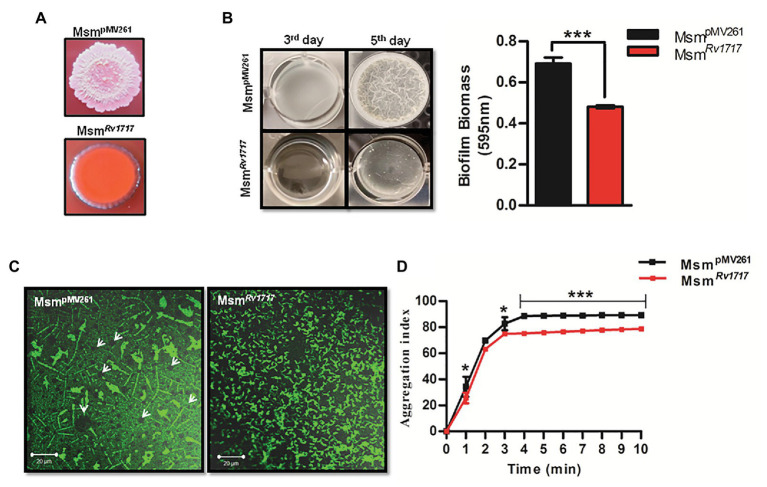
Rv1717 expression in *M. smegmatis* impairs biofilm growth and autoaggregation. **(A)** Colony color and morphology of Msm*^Rv1717^* and Msm^pMV261^ on Middlebrook (MB) 7H10 agar plates containing 100 μg/ml Congo red. **(B)** Pellicle formation by Msm*^Rv1717^* and Msm^pMV261^ at air-liquid interface of Sauton’s medium on third and fifth days. Biofilm biomass on day 5 was quantified using crystal violet (absorbance at 595 nm). **(C)** Confocal laser scanning microscopy image of biofilms stained with BacLight Green™ fluorescent stain obtained using 63× oil immersion objective. Arrow heads in left panel point to possible nutrient/water channels **(D)** Autoaggregation property of either strain measured as aggregation index (% fall in OD_600_), was monitored at 1 min intervals up to 10 min. Statistical significance of data wherever applicable is indicated by ^*^p < 0.05; ^***^p < 0.001. Data plotted are mean ± SD of three independent experiments.

Smooth vs. rough/wrinkled colony morphotype has been associated with the ability to autoaggregate and form pellicles (biofilms formed at the air-liquid interface) and biofilms (adherent biofilms) in several other bacteria (Römling et al., 1998; Friedman and Kolter, 2004; Uhlich et al., 2006). Congo red assay is a fast method to test biofilm formation on a solid media. This diazodye can bind to two major extracellular matrix components seen in different bacteria, namely amyloid and cellulose to produce brown and pink color, respectively (Cimdins et al., 2017). Congo red has been specifically used to stain the cellulose component of *M. tuberculosis* biofilms and was found to bind biofilms and not the planktonic bacilli (Trivedi et al., 2016). Msm^pMV261^ strain produced rough, wrinkled and pink colonies, while Msm*^Rv1717^* strain produced nearly round, smooth colonies which were orange with a pink periphery (Figure 4A). The smooth texture and aberrant Congo red staining of Msm*^Rv1717^* colonies suggests that the overexpression of Rv1717 in *M. smegmatis* is probably inhibiting biofilm matrix formation.

Further, the ability of the Msm*^Rv1717^* strain to form biofilm and pellicle on liquid media was compared to that of Msm^pMV261^ strain. Biofilms of either strain in Sauton’s medium on day 5 were quantified by crystal violet assay. Pellicle and biofilm formation by Msm*^Rv1717^* strain was significantly reduced (*p* < 0.001) as compared to Msm^pMV261^ strain (Figure 4B). Biofilm formation was also monitored at regular time intervals up to 96 h. A significant impairment (*p* < 0.001) in biofilm formation by Msm*^Rv1717^* was apparent by 40 h of growth (Supplementary Figure S5). Since the two strains show similar growth rate (Supplementary Figure S4), the reduced biofilm formation is due to impaired ability to form biofilm rather than slow growth.

Biofilms of either strain on day 5 were also studied by confocal imaging after staining with a fluorescent stain BacLight Green. Images showed that while Msm^pMV261^ displayed a structured biofilm containing clusters of bacteria with circular regions likely representing fluid channels as described for bacterial biofilms on surfaces (Davit et al., 2013). On the other hand, Msm*^Rv1717^* bacteria were scattered (Figure 4C). The results suggest that the overexpression of Rv1717 on the cell wall is probably inhibiting the formation of bacterial aggregates and/or biofilm matrix, eventually affecting biofilm development.

### Expression of Rv1717 in *Mycobacterium smegmatis* Reduces Autoaggregation Property

Cell-cell adhesion or autoaggregation is believed to precede maturation of biofilms and formation of pellicles in *Pseudomonas aeruginosa* (von Gotz et al., 2004) and mycobacteria (Yang et al., 2017; Clary et al., 2018; DePas et al., 2019). A positive correlation between autoaggregation and biofilm forming ability has been observed for *Sinorhizobium meliloti*, wherein both properties depend on synthesis of exopolysaccharides (Sorroche et al., 2012). Since the expression of Rv1717 caused impairment of *M. smegmatis* biofilms and pellicles, we tested the effect of the same on autoaggregation. The presence of aggregates in the bacterial suspension would lead to faster sedimentation of the aggregates resulting in a decrease in optical density (OD_600_) of the supernatant with time. The aggregation index (% fall in OD_600_) was calculated at 1 min intervals up to 10 min, for Msm*^Rv1717^* and Msm^pMV261^ strains. It was observed that the OD_600_ of Msm*^Rv1717^* dropped at a significantly lower rate compared to the Msm^pMV261^ strain resulting in a lower aggregation index (Figure 4D).

### Exogenous Rv1717 Inhibits Biofilm Formation and Disrupts Preformed Biofilms

**Figure 5:**
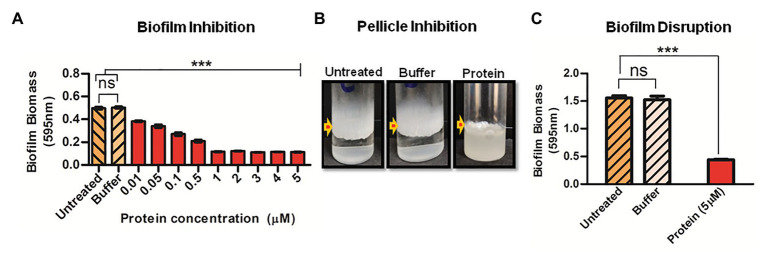
Exogenous Rv1717 protein inhibits biofilm formation and degrades biofilms. **(A)** Wild type *M. smegmatis* was allowed to form biofilm in Sauton’s medium containing different concentrations (0.01–5 μM) of purified Rv1717 protein in sodium phosphate buffer/buffer only/neither (untreated). On day 3, the biofilm biomass was quantified by crystal violet assay. **(B)** Fifth day air-liquid interface pellicle of *M. smegmatis* culture in Sauton’s medium in presence of 1 μM Rv1717. **(C)** *Mycobacterium smegmatis* biofilms were treated with 5 μM Rv1717 protein in sodium phosphate buffer pH 8.0 or buffer only. The biofilm biomass was quantified by crystal violet assay. Statistical significance of data wherever applicable is indicated by ^***^p < 0.001. Data plotted are mean ± SD of three independent experiments.

Since Rv1717 seems to localize to the poles rather than uniformly across the cell wall, we hypothesized that its function as a glycosyl hydrolase could be to cleave some polymer bridge that binds bacteria to each other (cohesion) or substratum (adhesion). Cohesion and adhesion are the initial stages of biofilm formation (Fletcher, 1980). We grew *M. smegmatis* (wild type) biofilms in presence of different concentrations of purified Rv1717 protein. On day 3, the biofilms were quantified by crystal violet assay. A continuous dose-dependent decrease in biofilm biomass was observed (Figure 5A). Effect of exogenous Rv1717 on pellicle formation was tested separately in borosilicate tubes. Rv1717 protein prevented pellicle formation at the air-liquid interface and bacterial cells grew exclusively as planktonic forms (Figure 5B). Therefore, exogenous Rv1717 is capable of inhibiting pellicle and biofilm formation, suggesting that it has an external substrate which is essential for the process.

If Rv1717 is degrading the exopolysaccharide that establishes adhesion and cohesion, it is expected to degrade the same in a biofilm. To assay for biofilm disruption, purified Rv1717 protein was added at a concentration of 5 μM to preformed biofilms of *M. smegmatis* (wild type). Sodium phosphate buffer was used as a negative control and biofilm biomass was quantified using crystal violet assay. Addition of Rv1717, but not the buffer, significantly removed the biofilm (Figure 5C).

### Galactose Binding Lectin Binds to Mycobacterial Biofilm Matrix or the Extracellular Polymeric Substance

Our enzymatic data of Rv1717 shows that it hydrolyses glycosidic bonds linking β-D-galactopyranose (β-D-Gal) or N-acetylgalactosamine (β-D-GalNAc). Interestingly, the enzyme was weakly active on pNP-β-D-galactofuranose, the form of glalactose present in the mycobacterial cell wall arabinogalactan (McNeil et al., 1987). Hence, the galactan in the cell wall is unlikely substrate of Rv1717. Exopolysaccharides form the major component in the EPS of bacterial biofilms. The *M. tuberculosis* EPS contains cellulose as the major exopolysaccharide (Trivedi et al., 2016). Cellulose is a glucose polymer containing β (1 → 4) glycosidic bonds. Presence of galactose containing exopolysaccharides in mycobacterial EPS has not been reported. *Pseudomonas aeruginosa* biofilm matrix contains either Pel, Psl, or alginate as the exopolysaccharide (Mann and Wozniak, 2012). Both Psl and Pel are indispensable for biofilm formation (Yang et al., 2011). Pel is composed of partially deacetylated N-acetyl-D-glucosamine (D-GlcNAc) and β-D-GalNAc that crosslinks eDNA in the biofilm matrix (Jennings et al., 2015). Pel is essential for initiating and maintaining cell-cell interactions in biofilms (Colvin et al., 2011; Yang et al., 2011) and under some conditions, plays a role in adherence of cells to a surface (Vasseur et al., 2005).

**Figure 6:**
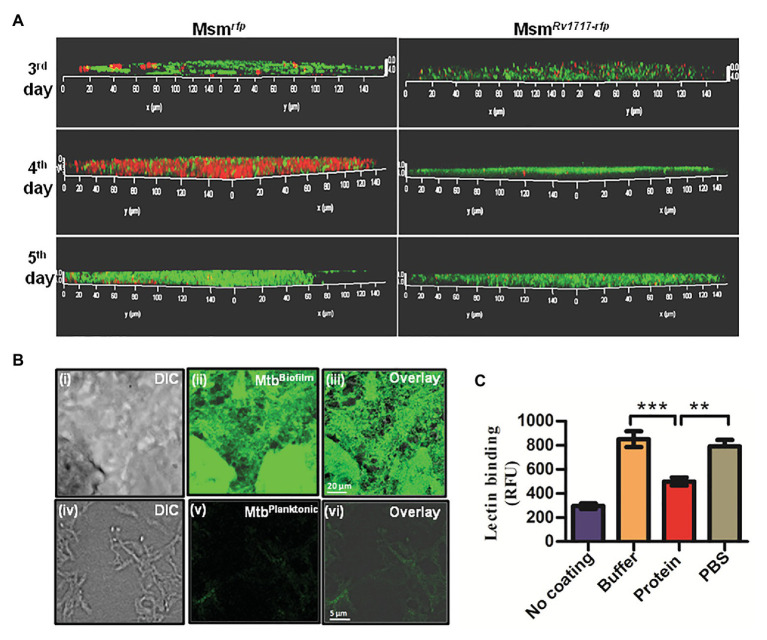
Rv1717 reduces the binding of WFL, a galactose-specific lectin to mycobacterial extracellular polymeric substance (EPS). **(A)** *Mycobacterium smegmatis* recombinant strains expressing RFP fused to Rv1717 (Msm*^Rv1717-rfp^* or RFP alone (Msm*^rfp^*) were grown in Sauton’s medium to form biofilms on coverslips. On day 3, 4, and 5, the coverslips were stained with Fluorescein-tagged *Wisteria floribunda* lectin (WFL) and subjected to confocal laser scanning microscopy. Imaging was done with a 100× water immersion objective laser line. The representative images show WFL as green and bacteria as red entities. Absence of fluorescence mixing (orange) indicates that WFL stains the EPS and not the bacteria *per se*. WFL binding was seen on all days and was progressive in case of both strains. Bacteria are overlaid with the EPS on fifth day and hence are barely visible. **(B)** Fluorescein-WFL stained biofilms (upper panel) and planktonic culture (lower panel) of Mtb H37Ra. **(C)** Exopolysaccharides were extracted and purified from *M. smegmatis* biofilms, coated on 96-well ELISA plates and treated with Rv1717 protein (12 μM) in buffer or buffer without the protein for 1 h at 37°C. Residual EPS after thorough washing was stained with Fluorescein-tagged WFL and quantified by fluorescence measurement. Data plotted are mean ± SD of three independent experiments. Statistical significance of data wherever applicable is indicated by ^**^p < 0.01; ^***^p < 0.001.

In order to detect any β-D-Gal/β-D-GalNAc residues that may be present in the mycobacterial EPS, we stained *M. smegmatis* biofilms with fluorescein labeled WFL. Lectins are proteins having strict specificity to monosaccharides or oligosaccharides. WFL binds specifically to N-glycans terminating in β-linked N-acetylgalactosaminides, especially those with β-D-GalNAc-[1.4]-D-GlcNAc) termini and to terminal galactose residues with lower avidity (Haji-Ghassemi et al., 2016). *Mycobacterium smegmatis* strains expressing RFP (Msm*^rfp^*) or Rv1717-RFP fusion (Msm*^Rv1717-rfp^*) were allowed to form biofilms on coverslips. Three, four, and five days-old biofilms were stained by fluorescein labeled WFL and imaged by a confocal laser scanning microscope. WFL staining was detected as green fluorescence, while bacteria as red fluorescence. Since there was no orange fluorescence detectable, it can be assumed that WFL is binding to the EPS and not bacteria (Figure 6A). WFL binding was seen on all days and was progressive in case of both strains. In case of Msm*^rfp^*, bacteria were to be seen all over the biofilm on day 4, but were hardly visible on day 5, probably because the bacteria were completely overlaid by EPS. EPS accounts for more than 90% of dry mass in most biofilms (Flemming and Wingender, 2010). The WFL fluorescence was more intense and thicker for Msm*^rfp^* than for Msm*^Rv1717-rfp^*. Moreover, the Msm*^Rv1717-rfp^* bacteria failed to form aggregates and hence were barely visible on all days.

*Wisteria floribunda* lectin was also found to stain Mtb (H37Ra) biofilm strongly, but not the planktonic bacilli (Figure 6B). Therefore, WFL binding to Msm and Mtb biofilms suggests the presence of β-D-GalNAc or terminal β-D-Gal residues in the exopolysaccharide. In addition, the reduced bacterial presence in *M. smegmatis* expressing Rv1717 in the biofilm suggests that Rv1717 is involved in preventing adhesion and/or bacterial cohesion.

### Rv1717 Degrades Extracted Exopolysaccharides of *Mycobacterium smegmatis* EPS and Decreases Its Binding to Galactose-Specific Lectin

In order to confirm that Rv1717 targets the galactoside linkages outside of the bacteria, i.e., the exopolysaccharides of the biofilm matrix (EPS), we tested the enzymatic action on extracted EPS from *M. smegmatis* biofilms. In a solid phase assay, purified EPS was treated with Rv1717 protein or buffer alone. After thorough washing, the residual EPS was stained with fluorescent WFL and fluorescence was estimated. The EPS showed significantly lower WFL binding after treatment with Rv1717, but not the buffer alone (Figure 6C). The results suggest that Rv1717 galactosidase is targeting the galactoside linkages in the exopolysaccharide component of the mycobacterial EPS.

### Rv1717 Helps Dispersal of Mtb Bacteria From the Biofilm

In general, in the biofilm mode of growth in bacteria is a cyclic process, which is initiated by the attachment of planktonic organisms to a surface, formation of aggregates (microcolonies), synthesis of EPS and finally a voluntary escape from the biofilm structure in a process referred to as “dispersion” (Guilhen et al., 2017; Rumbaugh and Sauer, 2020). Dispersion is a survival strategy of the biofilm bacteria to ensure their continued existence (Davies, 2011; Flemming, 2011). It has been suggested that extracellular Mtb in necrotizing lesions likely grows as biofilms (Basaraba and Ojha, 2017). Since Rv1717 has been shown to be expressed by Mtb in TB patient lungs (Garton et al., 2008; Walter et al., 2015), we hypothesized that Mtb may use Rv1717 to disperse from biofilms.

**Figure 7:**
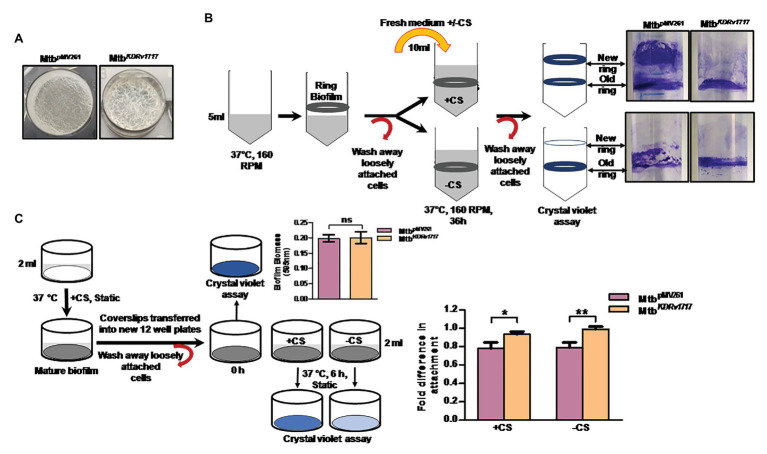
Rv1717 down regulation in *Mycobacterium tuberculosis* (Mtb) leads to impaired *in vitro* biofilm dispersion **(A)** Two weeks-old surface pellicles of Mtb^pMV261^and Mtb*^KDRv1717^* in Sauton’s medium. **(B)** Left panel: Schematic representation of the qualitative ring biofilm dispersal assay as described in methods. “+CS” represents medium with carbon source (glycerol) and “−CS” represents medium without carbon source. Right panel: shows ring biofilms of Mtb^pMV261^ and Mtb*^KDRv1717^* stained with crystal violet after 36 h of adding the fresh medium. **(C)** Schematic representation of quantitative microtiter plate assay to measure the biofilm dispersion as described in methods. Biofilm biomass before and 6 h after addition of induction medium with carbon source (+CS) or without (-CS) were quantified by crystal violet assay. Fold difference in biomass (OD_595_) was calculated as the ratio of OD_6h_ to OD_initial_. The y axis represents the fold difference. A value close to 1 (no change) indicates no dispersion. Values less than 1 (lower OD_6h_) indicate dispersion. Statistical significance of data wherever applicable is indicated by ^*^p < 0.05; ^**^p < 0.01.

Various environmental stresses have been reported to induce biofilm dispersion in other bacteria (Guilhen et al., 2017). Dispersion was measured by a ring biofilm dispersion assay as described by Stacy et al. (2016). The assay is based on the principle that bacteria form a ring biofilm when grown under shaking conditions, which under a specific stress would disperse. If the conditions are favorable for biofilm growth, the dispersed cells will form a new ring biofilm. The Rv1717 transcription in Mtb was down-regulated by ~50% using pMV261 vector constitutively expressing the anti-sense transcript (Supplementary Figure S6). We compared the dispersion of Mtb having Rv1717 expression downregulated (Mtb*^KDRv1717^*) with Mtb carrying vector alone Mtb^pMV261^. We used fresh medium or glycerol-free (carbon starvation) medium to induce dispersion of Mtb biofilms. Mtb*^KDRv1717^* formed biofilms similar to Mtb^pMV261^ (Figure 7A). When the biofilms were supplied with fresh complete medium, after 36 h, Mtb^pMV261^ strain formed a new ring biofilm above the previous one, indicating dispersion from the latter. Mtb*^KDRv1717^* did not form a new ring indicating that it was unable to disperse from the older one (Figure 7B, upper panel). However, when the biofilm was supplied with medium without the carbon source, neither strain formed new ring biofilm (Figure 7B, lower panel). This could be due to the fact that formation of biofilm requires a carbon source for bacterial multiplication and EPS biosynthesis. This method has the disadvantage that dispersion is confirmed only up on the formation of a new biofilm. Moreover, biofilms contain an undetermined fraction of dead cells (Cox et al., 2000). Since crystal violet stains even the dead cells along with the EPS, the first biofilm ring remains can still be seen even when the strain is capable of dispersion.

Hence, we used a more quantitative assay to estimate dispersion in which, the biofilm biomass on coverslips after 6 h induction of dispersion was quantified by crystal violet assay. Dispersed Mtb cells are less likely to form a new biofilm during this short time interval. Hence the crystal violet staining is expected to be solely due to the original biofilm. The biofilms of the knock down strain showed significantly higher biofilm biomass (lower dispersion) at 6 h post induction compared to the empty vector control strain (Figure 7C).

**Figure 8:**
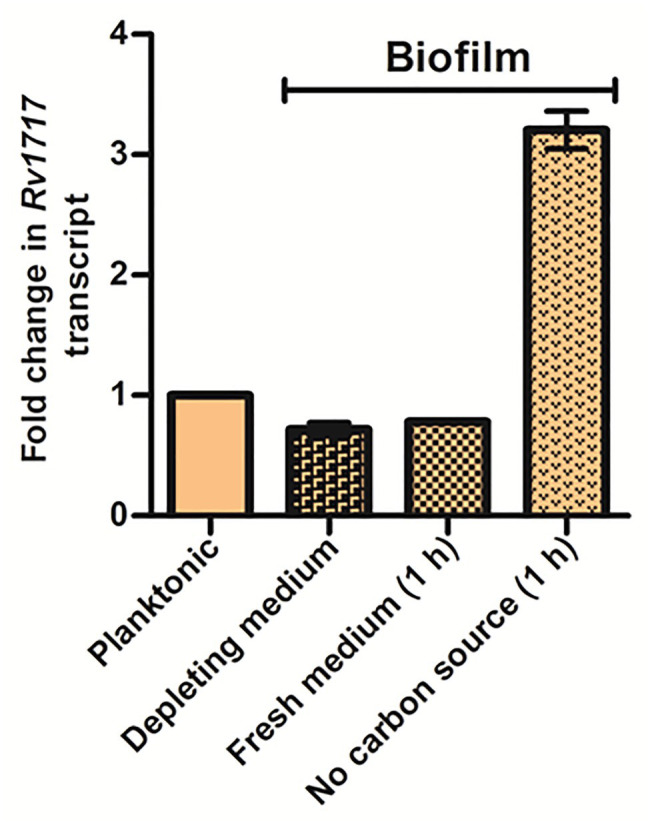
*Mycobacterium tuberculosis* biofilm upregulates *Rv1717* expression upon sensing a dispersal signal. Transcripts of *Rv1717* were estimated in a late log phase planktonic culture and biofilms of Mtb H37Rv wild type by quantitative RT-PCR. In case of biofilms, RNA was extracted before and after 1 h of replacing the used medium (depleting medium) with fresh medium with or without carbon source. Graph shows the linear fold change of *Rv1717* transcripts calculated by the 2^^▵▵Ct^ method using *SigA* transcripts for normalization. The values plotted are the mean ± SD of three biological replicates.

Next, we compared the *Rv1717* expression levels in wild type Mtb during planktonic growth, in biofilm and in dispersing biofilm by quantitative RT-PCR. Biofilm dispersion was induced by replacing the used medium with either fresh medium or medium without carbon source (carbon starvation). *Rv1717* transcripts were estimated in the biofilm, while in the used medium and 1 h after replacing the used medium and compared to the transcript levels in planktonic culture. Compared to the late log phase planktonic culture, biofilm bacteria had ~30% lower expression of Rv1717. Addition of fresh complete medium did not change the transcription significantly at 1 h. However, carbon starvation caused ~3-fold upregulation at 1 h (Figure 8), suggesting that carbon starvation is stronger trigger for bacteria to leave the biofilm matrix. The comparative transcript levels therefore suggest that Rv1717 is probably maintained throughout planktonic growth at a certain basal level, down regulated during biofilm growth and promptly upregulated when dispersal from the biofilm is imminent.

## Discussion

The present study was aimed to characterize the molecular and cellular functions of Rv1717, a conserved hypothetical protein of Mtb. Rv1717 has 100% identical genetic homologues in all Mtb genomes sequenced so far, as well as in *M. bovis*. Orthologues are also found in several non-tubercular mycobacteria and other Actinobacteria. In addition to phylum Actinobacteria, orthologues are present in Alpha-proteobacteria and Beta-proteobacteria. However, some pathogenic mycobacteria like the *M. avium* complex, *M. marinum*, and *M. leprae*, do not have orthologues. The gene has been reported to be nonessential for the *in vitro* growth of *M. tuberculosis* H37Rv (Sassetti et al., 2003; Griffin et al., 2011; DeJesus et al., 2017). Interestingly, the Rv1717 transcripts have been detected in TB patient sputa (Garton et al., 2008; Walter et al., 2015). The protein sequence has a cupin domain, named after its β-barrel shape (Dunwell 1998). Cupin domains are characteristic of a superfamily of proteins with extremely diverse functions. The members of this super family include metal independent, sugar binding non-enzymatic proteins as well as metal dependent enzymatic proteins (Uberto and Moomaw, 2013). In addition to the cupin domain, a GH motif was also detected, while the sequence was searched in the KEGG database. The Carbohydrate Active Enzymes database or CAZy database currently lists 31 proteins encoded by the Mtb H37Rv genome as GHs, a small subset of which has been biochemically characterized. Rv1717 is not there in the CAZy list of carbohydrate active protein families for Mtb H37Rv genome. Despite that, we performed a multiple sequence alignment of Rv1717 amino acid sequence with CAZy GH families that possess a β-jelly roll structure composed of β-barrel strands, similar to the cupin domain present in Rv1717. No significant homology was identified with the active site motifs of any of these families. Site directed mutagenesis studies are required to identify the active site residues of this newly identified GH member.

We first tested the GH activity of purified recombinant Rv1717 protein. We show that Rv1717 is a beta-galactopyranosidase, which recognizes the β-glycosidic bonds formed with β-D-Gal or β-D-GalNAc. Rv1717 was specific for the pyran form of galactose rather than the furan form and also β-galactoside and not the α-galactoside. It is worth mentioning here that in the mycobacterial cell wall arabinogalactan, both galactose and arabinose are present in the furan forms (Gal*f* and Ara*f*), which appear infrequently in nature (McNeil et al., 1987). The finding that Rv1717 is a β-D-galactosidase specific to pyran ring form of galactose suggests that its physiological substrate is probably not the galactan chains in the mycobacterial cell wall.

In order to get insights into the cellular function of Rv1717, the gene was expressed in non-pathogenic fast growing surrogate, *M. smegmatis*, which lacks an ortholog of the protein. The expression of Rv1717 caused changes in the cell wall associated properties as well as colony morphotype. *Mycobacterium smegmatis* expressing Rv1717 was sensitive to cell wall perturbing agent SDS and showed altered cell wall permeability. The colonies of the recombinant strain were rounder and smoother than the strain carrying empty vector. Colony morphotype change from rough to smooth has been reported to be linked with biofilm formation in other bacteria (Römling et al., 1998; Friedman and Kolter, 2004; Uhlich et al., 2006). Congo red is an amphiphilic dye that has been used more often for a qualitative assessment of the overall hydrophobicity of the mycobacterial cell wall (Jankute et al., 2017). Cell wall hydrophobicity in turn promotes bacterial aggregation and biofilm formation in liquid cultures (Arora et al., 2008; Kundu et al., 2017). However, in other bacteria Congo red staining is employed as a faster screening assay for biofilm properties since it stains the major biofilm matrix components namely amyloid and cellulose to produce brown and pink color, respectively (Cimdins et al., 2017). In mycobacteria, Trivedi et al. (2016) used Congo red to specifically stain cellulose present in thiol stress-induced *M. tuberculosis* biofilms as the dye binds to (1–4)-β-D-glucopyranosyl units with strong affinity. In the present study, Congo red staining produced very obvious differences between the Msm strains based on the expression of Rv1717. The characteristic pink staining of cellulose by Congo red was limited to the periphery of Msm*^Rv1717^* colonies. Therefore, increased uptake of hydrophilic dye EtBr and reduced uptake of lipophilic Nile red point toward decreased hydrophobicity of Msm*^Rv1717^* that could impact its ability to form aggregates and biofilms. Altered Congo red staining of Msm*^Rv1717^* colonies can also be interpreted as impaired formation of colony biofilm and hence poor staining of cellulose in the EPS. Biofilm and pellicle formation in liquid cultures further confirmed the observations on Congo red solid media.

In the biofilm mode of growth, microorganisms including certain yeasts, live attached to a substratum, as structured communities encased in a self-produced polymeric matrix also known as the EPS (Costerton et al., 1995, Costerton, 1999). The EPS is comprised of polysaccharides, proteins, eDNA, lipids, and other molecules. Cellulose has been identified as the major exopolysaccharide of Mtb biofilms induced *in vitro* by thiol stress (Trivedi et al., 2016). Subcellular localization of Rv1717 in *M. smegmatis* revealed that the protein localizes on the cell wall and notably to the poles. The absence of signal sequence motifs for Sec, Tat, lipoprotein, or ESX protein secretion systems suggests that the protein reaches the cell surface through a non classical secretion pathway. Next, we hypothesized that, being a GH on the cell wall that affects the biofilm formation, Rv1717 could be interfering with the EPS structure and thereby preventing cell-cell as well as cell-substratum adherence. Subsequently, it was observed that addition of purified Rv1717 protein to *M. smegmatis* cultures inhibited not only biofilm and pellicle formation but also degraded preformed biofilms. This observation supported our hypothesis that the enzymatic substrate of Rv1717 probably lies outside of the mycobacterial cell.

Cellulose is a polymer of glucose containing β (1 → 4) glycosidic bonds, while Rv1717 specifically cleaves glycosidic bonds formed with β-D-Gal or β-D-GalNAc. However, galactose was not been detected in the GC-MS profile of *M. tuberculosis* EPS (Trivedi et al., 2016). But it is an essential component of EPS in several other genera, such as *Pseudomonas* (Ma et al., 2007), *Bacillus* (Chai et al., 2012), *Lactobacillus* (Lebeer et al., 2009), and *Bradyrhizobium* (Quelas et al., 2010). Galactose has been detected in the EPS of all ESKAPE pathogens and toxigenic strain of *E. coli* as the second most abundant sugar after mannose (Bales et al., 2013). GalNAc is found in EPS of diverse bacteria such as *P. aeruginosa* (Jennings et al., 2015), *Desulfovibrio vulgaris* (Zhu et al., 2018), *Bacillus subtilis* (Purish et al., 2013), and cyanobacteria (Zippel and Neu, 2011). Pel, the primary biofilm exopolysaccharide of *P. aeruginosa* PA14 strain (Colvin et al., 2012), is composed of partially acetylated 1 → 4 glycosidic linkages of D-GalNAc and D-GlcNAc (Jennings et al., 2015). Pel crosslinks eDNA in the biofilm stalk through ionic interactions (Jennings et al., 2015). The pathogenic fungus *Aspergillus fumigatus* produces a biofilm matrix consisting of galactosaminogalactan, a cationic polymer of α-1,4-linked galactose and partially deacetylated D-GalNAc (Le Mauff et al., 2019). We used the WFL, which binds specifically to N-glycans terminating in β-D-GalNAc and terminal galactose residues (Haji-Ghassemi et al., 2016), to demonstrate that *M. smegmatis* and Mtb EPS does contain one of these. The fluorescent labeled lectin did not stain the bacteria, suggesting that the galactose moieties being bound exist in the matrix and not the cell wall. Since galactose was not detected by GC-MS in *M. tuberculosis* EPS in the previous study (Trivedi et al., 2016), the WFL-binding could be due to the presence of β-D-GalNAc, which would produce a peak at a lower retention time. More accurate structural studies of *M. tuberculosis* EPS are required to support our findings.

One or multiple species of microbes entrapped in the EPS are tolerant to both antimicrobials and host defenses (Costerton, 1999; Leid et al., 2009). This makes biofilm life style the preferred mode of growth of several microorganisms both in the environment and host. Biofilm formation is lately documented as a cyclic process initiated by planktonic organisms attaching to a surface, forming aggregates (microcolonies), synthesis of EPS and finally escaping from the biofilm structure in a process referred to as “dispersion.” The biofilm dispersion is an active, voluntary escape of bacteria from the biofilm macrostructure to spread to new locations (Guilhen et al., 2017; Rumbaugh and Sauer, 2020). Dispersion, most likely, is a survival strategy of the biofilm bacteria to escape the deteriorating biofilm environment and ensure its continued existence (Davies 2011). Moreover, biofilm dispersion is necessary for bacterial dissemination of several pathogenic bacteria *in vivo* (Guilhen et al., 2017). Published work in other bacteria suggest that biofilm dispersion starts with sensing of cues (self/environmental), followed by intracellular signaling that leads to a fall in intracellular c-di-GMP and ends by disintegration of the biofilm matrix by various effectors (Karatan and Watnick, 2009; Guilhen et al., 2017; Rumbaugh and Sauer, 2020). Matrix disintegration hence coincides with the increased expression and production of matrix-degrading enzymes such as glycosyl hydrolases (Kaplan et al., 2003, 2004; Yu et al., 2015; Cherny and Sauer, 2020), DNases (Whitchurch et al., 2002; Mann and Wozniak, 2012; Cherny and Sauer, 2019) and proteases (Boles and Horswill, 2008; Gjermansen et al., 2010), among other mechanisms.

Since polysaccharides are the major and common component of microbial biofilms, the role of glycosyl hydrolases in biofilm dispersion is now well recognized for various bacteria and fungi. Examples are Dispersin B of *Actinobacillus actinomycetemcomitans* (Fekete et al., 2011), PelA and PslG of *P. aeruginosa* (Yu et al., 2015; Baker et al., 2016), PgaB of *E. coli* and *Bordetella* spp. (Little et al., 2018) and Sph3 of the fungal pathogen *A. fumigatus* (Le Mauff et al., 2019). α-amylase and cellulase, both GHs, have been demonstrated to effectively disrupt *Staphylococcus aureus* and *P. aeruginosa* monoculture and coculture biofilms (Flemming et al., 2016). Some of the biochemically characterized glycosyl hydrolases of Mtb are involved in α-glucan metabolism, β-glycan metabolism, α-demannosylation and peptidoglycan remodeling (van Wyk et al., 2017). The confirmed and presumed β-glycanases of Mtb include cellulases Rv0062 (Varrot et al., 2005) and Rv1090 (Mba Medie et al., 2011), β-glucanase (Rv0186), β-xylanase (Rv3096) and the catalytically inactive β-1,3-glucanase (Rv0315; Dong et al., 2015). The secreted β-glycanases are yet to be demonstrated to have any role in biofilm matrix remodeling or degradation.

After confirming the degradation of mycobacterial EPS by Rv1717, its role in Mtb biofilm dispersion was tested using Mtb strain with reduced expression of Rv1717 (knock-down strain). Biofilm formation was not affected in the knock-down strain, however, when induced to disperse, the knock down strain showed significantly lower dispersion compared to the wild type. This observation suggests that Mtb most likely uses Rv1717 during its biofilm cycle in order to disperse from a deteriorating biofilm. Mtb biofilms exposed to carbon starvation also showed a sudden transcriptional upregulation of *Rv1717*, further supporting the biofilm dispersion findings. Indirectly, this finding, along with the previous sputum bacilli transrciptome studies (Garton et al., 2008; Walter et al., 2015), favors the notion that the pathogen does make biofilm in the host tissue (Basaraba and Ojha, 2017).

## Data Availability Statement

The original contributions presented in the study are included in the article/Supplementary Material, further inquiries can be directed to the corresponding author.

## Author Contributions

SB, RM, and MK designed the experiments. SB, RM, UV, and RS performed the experiments. SB, RM, MA, and MK analyzed the data. SB and MK wrote the manuscript draft. All authors reviewed, edited and approved the manuscript.

### Conflict of Interest

The authors declare that the research was conducted in the absence of any commercial or financial relationships that could be construed as a potential conflict of interest.
