# Supplementary material for: Rv1717 Is a Cell Wall - Associated β-Galactosidase of Mycobacterium tuberculosis That Is Involved in Biofilm Dispersion
Source: Front Microbiol. 2021 Jan 15;11:611122. doi: 10.3389/fmicb.2020.611122 (PMC7873859; doi:10.3389/fmicb.2020.611122)
Supplement: Supplementary file 1 [file Data_Sheet_1.pdf]

Figure:S1

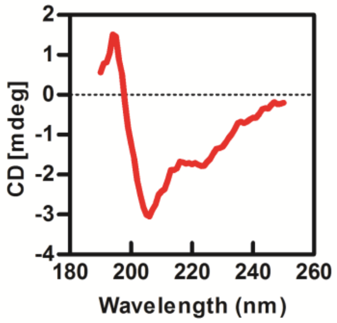

Figure:S2

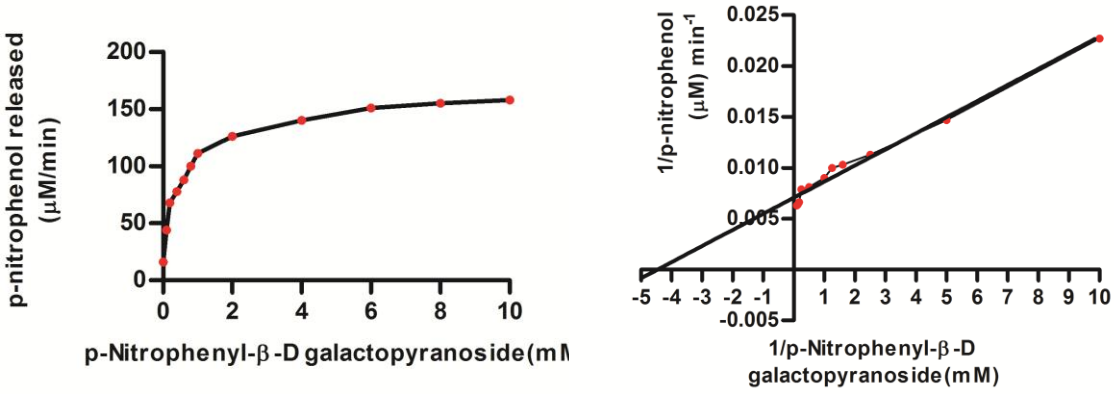

Figure:S3

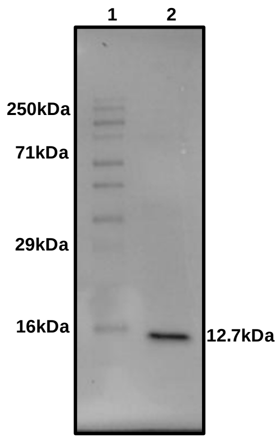

Figure:S4

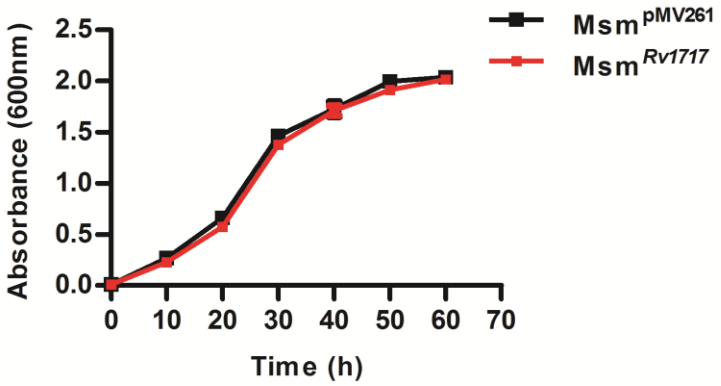

Figure:S5

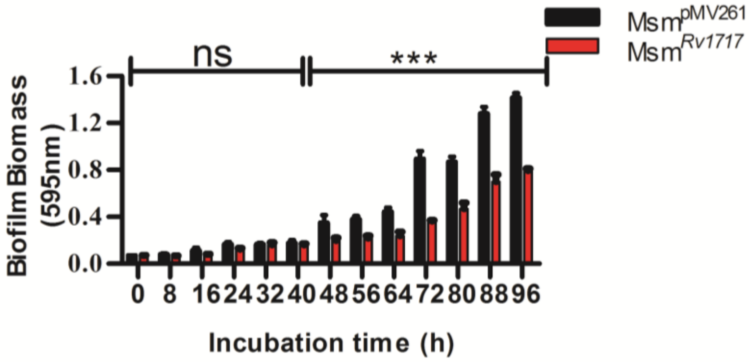

Figure:S6

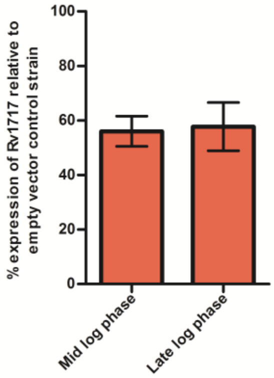

S1-Table

Primers used for expression of Rv1717 in *E. coli* (C41) expression strain

NheI1717F	CTAGCTAGCATGAAACTGACACGAGCGTCGCAG
XhoNSC1717R	CCGCTCGAGTGCCACCTCTGCAACCG

Primers used for overexpression construct in pMV261

$Rv1717^{HisTag}F$	CCGGAATTCATGAAACTGACACGAGCGTCGCAGGCCCCCAGGTATGTG
$Rv1717\ ^{HisTag}R$	CCCAAGCTTTCAGTGGTGGTGGTGGTGGTGCGCTACCTCGGCAACCGG

Primers used for Red fluorescent reporter construct

1717+lin-F	CGAGAGTAGGAATTCATGAAACTGACACGAGCGTCG
1717+lin-R1	CGCCGAGCCCGCCGAACCCGCCACCTCAGCAACCG
1717+lin-R2	AAATTCACCTGACCCCGCCGCCGAGCCCGCCGAACC
Fred-Fn	GGGTCAGGTGAATTTATGGTCTCGGAACTCATTAAGGAAAATATG
Fred-R	GACATCGATAAGCTTTCATTTGCCGCCATCG

Primers used for Knock down construct in pMV261

KD1717F	CCCAAGCTTATGAAACTGACACGAGCG
KD1717R	CCGGAATTCTCACGCCACCTCTGCAAC

Primers used to confirm Knock down strain

qKD1717F	ATGAAACTGACACGAGCGTCG
qKD1717R	CCCACCCAGAATCGCTCGGT
SigA_F	GTGACCCGGGAACGCAT

SigA_R	GTGAGCGGCTCGGATGG

S1. Table. List of primers used in the study

SUPPLEMENTARY FIGURE CAPTIONS

Figure: S1 Normalized far-UV CD spectrum of Rv1717 protein recorded in phosphate buffer (pH 8.0) containing 100 mM NaCl. Spectrograms were averaged of three scans.

Figure: S2 Determination of K_m and V_{max} values for pNP-β-D-gal substrate (A) Michaelis-Menten enzyme kinetics of Rv1717 **(B)** Double-reciprocal plot of the initial velocities against pNP-β-D-gal. The values are average of three experiments.

Figure: S3 Western blot analysis of expression of Rv1717 in *M. smegmatis*: Expression of Rv1717 with a C-terminal 6×His tag in *M. smegmatis* was confirmed by anti-6×His tag antibody.

Figure: S4 Growth curve of Msm^{pMV261} and Msm^{Rv1717} strains in MB7H9 broth supplemented with OADC, glycerol, tween 80 and kan. Data are representative of mean±SD of three independent experiments.

Figure: S5 Biofilm formation kinetics. Msm^{Rv1717} and Msm^{pMV261} strains were grown in Sauton's medium in 96 well plates up to 4 days. Biofilm biomass was quantified by crystal violet assay at various time points as indicated. Data are representative of mean±SD of three independent experiments.

Figure: S6 Transcript analysis of *Rv1717* by quantitative reverse transcriptase PCR (qRT-PCR). Total RNA was extracted from mid-log or late log phase cultures of

Mtb^{pMV261} or Mtb^{KDRv1717} in supplemented MB7H9 broth. *Rv1717* transcripts were estimated by quantitative RT-PCR. Percentage change in Rv1717 transcripts in Mtb^{KDRv1717} was calculated relative to the empty vector control strain (Mtb^{pMV261}) by the 2 ^ (-△△ Ct) method using SigA transcripts for normalization. The values plotted are the mean±SD of three biological replicates.
